# DeCAF—Discrimination, Comparison, Alignment Tool for 2D PHarmacophores

**DOI:** 10.3390/molecules22071128

**Published:** 2017-07-06

**Authors:** Marta M. Stepniewska-Dziubinska, Piotr Zielenkiewicz, Pawel Siedlecki

**Affiliations:** 1 Institute of Biochemistry and Biophysics, Polish Academy of Sciences, Pawinskiego 5a, 02-106 Warsaw, Poland; martasd@ibb.waw.pl (M.M.S.-D.); piotr@ibb.waw.pl (P.Z.); 2 Department of Systems Biology, Institute of Experimental Plant Biology and Biotechnology, University of Warsaw, Miecznikowa 1, 02-096 Warsaw, Poland

**Keywords:** molecule representation, ligand-based screening, pharmacophore, bioactivity prediction

## Abstract

Comparison of small molecules is a common component of many cheminformatics workflows, including the design of new compounds and libraries as well as side-effect predictions and drug repurposing. Currently, large-scale comparison methods rely mostly on simple fingerprint representation of molecules, which take into account the structural similarities of compounds. Methods that utilize 3D information depend on multiple conformer generation steps, which are computationally expensive and can greatly influence their results. The aim of this study was to augment molecule representation with spatial and physicochemical properties while simultaneously avoiding conformer generation. To achieve this goal, we describe a molecule as an undirected graph in which the nodes correspond to atoms with pharmacophoric properties and the edges of the graph represent the distances between features. This approach combines the benefits of a conformation-free representation of a molecule with additional spatial information. We implemented our approach as an open-source Python module called DeCAF (Discrimination, Comparison, Alignment tool for 2D PHarmacophores), freely available at http://bitbucket.org/marta-sd/decaf. We show DeCAF’s strengths and weaknesses with usage examples and thorough statistical evaluation. Additionally, we show that our method can be manually tweaked to further improve the results for specific tasks. The full dataset on which DeCAF was evaluated and all scripts used to calculate and analyze the results are also provided.

## 1. Introduction

One of the outstanding challenges in virtual screening is the development of a fast and robust algorithm to compare many compounds and identify subsets with biological activity. It is often desired that such subsets are diverse with respect to their basic molecular scaffolds. Such scaffold hopping can be used to break out of the protected “patent space” or to find molecules with different, more desirable pharmacological properties.

All the above have prompted the development and application of ligand-based techniques that rely on a set of active/inactive ligands [1]. One highly popular idea is to represent a compound as a vector with the presence (1) or absence (0) of a set of features describing chemical structure and properties. This method is very efficient as both vector generation (fingerprint) and screening task (vector comparison) are computationally inexpensive. Unfortunately, fingerprint representations have very limited capacity, do not scale well with increasing compound size and complexity, depend strongly on a set of predefined features, and have poor scaffold-hopping performance [2,3].

As the affinity of a ligand-receptor interaction depends on atomic interactions in all three dimensions, alternative approaches incorporating 3D features have emerged. Unfortunately they introduce ligand flexibility into the equation, which is computationally much more expensive and error-prone primarily due to insufficient conformational sampling. Methods to overcome this problem have been proposed. One idea was to represent the 3D molecule as a set of absolute, invariant molecular fragments generated from 2D topologies [4]. This approach, focusing more on representation consistency rather than realism, was successfully applied for ligand alignments in CoMFA (Comparative Molecular Field Analysis) [5].

Other methods have been proposed, with shape-based representations of small molecules [6,7], molecular interaction field-based representations [8] or surface-based representations [9], among others. Although the computation time is decreased compared to the simple atomic distance-based methods, they still require generating multiple conformations (5–50 per ligand) for all compounds in the dataset [10].

One way to avoid conformational search, yet still describe the shape of a compound, is to represent the molecule’s connectivity, rather than its particular conformation. Such representations are closely related to molecular graphs, typically used to visualize chemical compounds. Among the examples of graph-based approaches are reduced graphs [11] and extended reduced graphs (ErG) [12]. However, these methods use graphs only as an intermediate stage and calculates a set of descriptors characterizing their composition and topology, which are then used to compare molecules.

In this work, we went a step further and developed a method that utilizes a graph as a final representation of a molecule. Although such an approach is computationally more challenging, graphs were successfully used to compare molecules, using both 2D and 3D structures [13,14], 3D pharmacophores [15], or even protein binding sites [16]. 3D methods, however, relay on pre-generated ligand conformations, which are computationally expensive and can influence results significantly.

In this work, we use a graph to describe the 2D structure of a molecule. As features, we utilize the pharmacophoric properties of atoms, which express the binding potential of a molecule and are more general than functional groups. We complemented this representation with a graph alignment procedure that allows for comparing and combining of multiple models. Our approach was implemented as a customizable Python module called DeCAF (Discrimination, Comparison, Alignment tool for 2D PHarmacophores) that can be easily combined with Open Babel or RDKit to facilitate ligand-based drug design.

## 2. Materials and Methods

DeCAF source code, documentation and installation instructions are publicly available at http://bitbucket.org/marta-sd/decaf. All data and results presented in this study were created and analyzed using Python (v. 2.7) [17].

To generate and compare different fingerprints in the SEA benchmark (see Section 3.2), we used Open Babel (development version from 30.08.2015) [18]. Open Babel was also used to read molecules and automatically convert them to DeCAF pharmacophore models. Ligand-based virtual screening benchmark (see Section 3.1) was downloaded from Github as a git repository [19] and run with RDKit (version 2016.03.2) [20]. All other data and Python scripts used in our study can be downloaded from http://bitbucket.org/marta-sd/decaf-supplementary. The repository also contains instructions on how to prepare a Python environment with all packages needed to reproduce our results and repeat our analysis.

### 2.1. Representation

**Figure 1:**
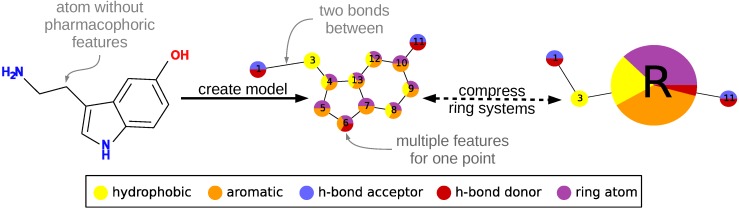
An example of creating a pharmacophore model from a single molecule. Atoms are converted into nodes and labeled with pharmacophoric features. During alignment, ring features are used to reduce the model to improve performance. Nodes numbering in the graph visualization corresponds to atom numbering.

To describe a molecule, DeCAF substitutes its functional groups with pharmacophoric points (see Figure 1), hence the “F” in the program’s name. Anything you can define with a SMARTS (SMiles ARbitrary Target Specification) pattern can be a feature and there are 5 elementary pharmacophoric points already implemented in DeCAF:(i) hydrophobic (“HH”); (ii) aromatic (“AR”); (iii) hydrogen bond donor (“HD”); (iv) hydrogen bond acceptor (“HA”); and (v) ring atoms (“R”) . The ring feature allows for the simplification of the alignment procedure (see next section and Supplementary Methods) and provides a natural penalty for aligning rings to aliphatic fragments of a molecule.

Each point and each of its features has a weight (the default is 1.0), which expresses the importance of that part of the model or its frequency, if the model was generated from multiple molecules. Nodes are organized into an undirected graph, in which atoms are connected to their direct neighbors or to the next atom in the molecule, if a neighbor is not present in the graph.

No calibration of the weighting scheme was performed as DeCAF aims to be applicable to a broad spectrum of chemical space. However, the pharmacophores can be manually tuned to incorporate the expert knowledge into the model. Weights can be changed to differentiate between crucial and less significant features. Edges can be added or removed to modify the distance constraints. In this study, DeCAF was tested by screening multiple targets without any target-specific adjustments. For some of the targets, we show how incorporating external knowledge can improve screening results (see Section 4).

### 2.2. Alignment

To compare two models, DeCAF aligns them by finding their maximal common subgraph. This is a common problem in cheminformatics, to which a solution was presented by Barrow and Burstall [21]. Similarly to their standard solution, DeCAF generates a modular product of two graphs and then searches for the best maximal clique in this product, which corresponds to the highest-scoring common part of two models.

The maximal common subgraph problem is NP-hard, and no polynomial algorithm for solving it is known. DeCAF uses additional simplifications and constraints to develop a fast, heuristic algorithm for finding a common part of two models. In this section, we will sketch the algorithm, and a more detailed description can be found in the Supplementary Methods.

The alignment procedure is divided into two phases. During the first phase, a coarse-grained version of each model is generated, in which rings and ring systems are compressed to single nodes using the pharmacophore “R” feature described above. Then, DeCAF generates their modular product and finds all maximal cliques within it using the Bron–Kerbosch algorithm with pivoting [22,23]. We select a clique with the highest similarity score, which corresponds to the best alignment of the reduced models.

One important modification to the Barrow and Burstall algorithm is that we introduce an additional constraint when adding edges to the modular product: we connect only those pairs whose corresponding nodes are in similar distance in both models. This decreases the number of edges in the modular product and therefore reduces the number of cliques. It also prevents DeCAF from producing alignments that do not preserve distances between features.

By default, DeCAF does not make any assumptions about the data. It requires a strict match between the distances in the compared molecules. With additional knowledge of the receptor and its ligands, it is possible to increase the accepted distance to better describe the target’s preferences. However, for a study with diverse targets, it might lead to false positive results. Therefore, only default parameters have been used. We also show an example of the target with less strict distance preference in the Section 4.

The result of the first phase of the alignment procedure is a coarse-grained alignment, which is an approximation of the actual comparison of the two original models. In the second phase of the algorithm, nodes that form rings are aligned with respect to the already aligned parts of the pharmacophores, resulting in a fine-grained alignment. This final alignment can then be used to calculate the similarity between the two models. DeCAF can also combine them into a single pharmacophore model using shared nodes as a core of the new model. To assess similarity between the models, DeCAF uses Dice similarity score for non-binary. The similarity score of models P and Q is given as:

$$S\left(P,Q\right)=\frac{S_{A}\left(P,Q\right)}{\sum_{i\in P}w_{i}+\sum_{j\in Q}w_{j}}=\frac{\sum_{(i,j)\in A}\sigma \left(n_{i},n_{j}\right)\left(w_{i}+w_{j}\right)}{\sum_{i\in P}w_{i}+\sum_{j\in Q}w_{j}},$$

where:

$S_{A}\left(P,Q\right)$ is a score for an alignment *A*,
$\sigma (n_{i},n_{j})$ is a similarity between nodes *i* and *j*, and
$w_{i}$ is a weights of node *i*.

For each pair of aligned nodes, DeCAF computes $\sigma (n_{i},n_{j})$, which is the ratio of their common features by the number of all features, multiplied by the sum of their weights ($w_{i}+w_{j}$). $S_{A}\left(P,Q\right)$ is the sum over those values and serves as a score for a given clique during the alignment procedure. It is also used to compute the final similarity score S(P,Q): $S_{A}\left(P,Q\right)$ is divided by the sum of weights of all features present in the two models. $S_{A}\left(P,Q\right)$ is a counterpart of the doubled number of common features in the binary version of Dice’s similarity scores, while the sum of all weights is an analogue to the number of all features.

### 2.3. Benchmark Preparation and Dataset Development

Although there are ready-to-use datasets available, most of them are designed for structure-based screening. For example, in the DUD-E (Directory of Useful Decoys, Enhanced) database, “decoys” (compounds assumed to be inactive) are chosen to have similar physicochemical properties to active compounds, but dissimilar structures, so that binding to the target would be highly unlikely. This makes them very easy to distinguish with ligand-based methods, which use structural similarity. Hence, we tested our method on two ligand-based benchmarks: screening benchmark for fingerprints provided by Riniker and Landrum [24] and a benchmark using SEA as the screening method based on a Lounkine et al. study [25].

The dataset used in the first benchmark was provided by the authors and consisted of 88 targets from MUV (Maximum Unbiased Validation), ChEMBL, and DUD (Directory of Useful Decoys) databases. The data, together with scripts running the benchmark, were downloaded from Github [19]. Scripts were adapted for DeCAF: molecule representation and similarity function were added to fingerprint_lib.py and scoring_functions.py, respectively. In addition, the main script—calculate_scored_lists.py—was parallelized.

The dataset for the second benchmark was not available, thus we reconstructed it using the description and supplementary files from the original work by Lounkine et al. For each of 73 targets, we created an extensive set of its potentially bioactive ligands by retrieving activity data from the ChEMBL database [26] and selecting ligands with $IC_{50}$, $EC_{50}$, $K_{d}$ or $K_{i}$ values below 1000 nM (accessed 10.2015).

**Figure 2:**
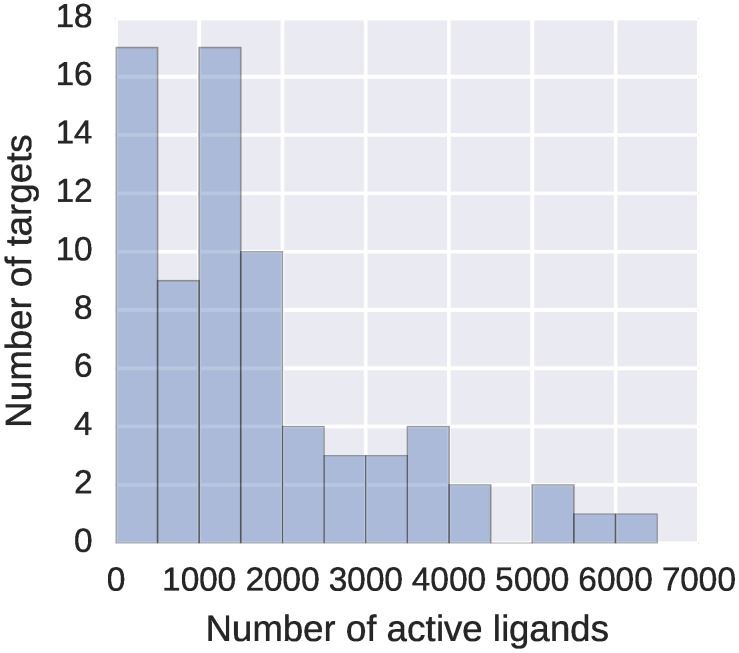
Number of active ligands in the SEA based activity prediction dataset. The dataset consists of 73 targets from diverse protein families, with a mean of 1680.05 active ligands per target (SD: 1438.91).

From this dataset, we filtered out all peptides and peptide-like compounds with more than 20 amino acids. Including longer peptides or other polymers, for which the overall 3D conformation defines their properties, would only slow down the computations but not improve the activity predictions – neither DeCAF, nor fingerprints would represent them properly. Final refined dataset consisted of 73 targets, with 59 to 6229 high affinity ligands describing each target (see Figure 2).

To test the models, data regarding interactions between 656 drugs tested by Lounkine et al. and the 73 targets were constructed. The validation set consists of: (i) low affinity (<30,000 nM) drug-target pairs from ChEMBL; (ii) those not present in ChEMBL, but reported by Lounkine et al. in their study; and (iii) present in the DrugBank database. All drug-target pairs with even lower affinity or without any data about interaction were assumed to be inactive.

For detailed description of each drug and target, see Supplementary file 1 and the original work by Lounkine et al.

SEA source code was also not available. We therefore implemented it based on the description provided in References  [25,27] (for more details, see Supplementary Methods and the repository at http://bitbucket.org/marta-sd/decaf-supplementary).

## 3. Results

### 3.1. Classical Similarity-Based Screening

DeCAF was evaluated with an open-source benchmarking platform created by Riniker and Landrum. This allowed for the direct comparison between DeCAF and 14 different fingerprints evaluated previously on this benchmark [24]. The benchmarking platform provides implementation of a simple ligand-based virtual screening, in which activity of a new compound is assessed based on its highest similarity to the active ligands for a given target.

**Figure 3:**
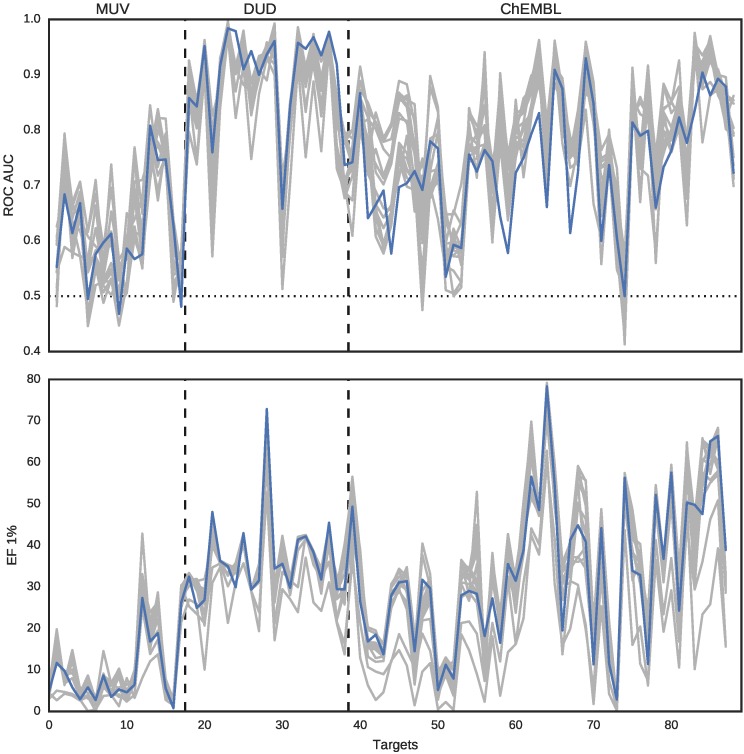
ROC AUC values (top panel) and EF 1% (bottom panel) for DeCAF (blue line) and 14 fingerprints (gray lines). The three data sources are separated with dashed lines. The baseline ROC AUC (0.5, results obtained for random predictions) is plotted with dotted line.

We compared 15 representations (14 fingerprints and DeCAF) with ROC AUC and enrichment factor (EF) for 1% of the top ranked predictions. Figure 3 shows that DeCAF performance is overall comparable to 14 fingerprints. Similarly to Riniker and Landrum, we conclude that variability between the targets is much higher than between the methods. It is worth noting that DeCAF has good early enrichment. There were no results for a smaller fraction of the database provided, but we included EF 0.25% in our second benchmark (see the next section).

### 3.2. SEA Screening

Besides classical similarity-based screening, DeCAF performance was also tested on a benchmark using SEA (Similarity Ensemble Approach) [25,27]. This method uses a more sophisticated approach to compare molecules to known ligands and better accounts for randomly occurring similarities. A more sophisticated definition of similarity not only reduces the number of false positives, but also improves scaffold hopping.

Briefly, the SEA predicts activity of a molecule against a given target by computing the overall similarity between the said molecule and the known target’s ligands. It is important to note that such datasets may differ in size and structural diversity. Therefore, the final result is expressed with *Z*-scores (standard score; distance between the obtained score and the mean, expressed in standard deviation units) and *E*-values (expected number of results as extreme as the observed one for randomly distributed data). *Z*-scores and *E*-values are complementary, but highlight different aspects of obtained results. A high *Z*-score indicates that there is a strong relationship between the new molecule and the set of ligands. *E*-value close to 0 shows that this relationship would not be found purely by random chance. Representing predictions this way allows to directly compare results from targets with varying number of ligands. It also enforces that the score has the same meaning regardless of the ligand-target pair used to calculate it. Therefore, in this study, we treat the whole dataset as a single screening panel and all presented results (ROC curves, enrichment factors and reliability analysis) are calculated globally. In addition, we also show comparisons with classical fingerprints implemented in Open Babel and USRCAT. Full details on the comparison are provided in the Supplementary Methods.

#### 3.2.1. Comparison to Fingerprints

To avoid bias, we compared DeCAF with all four fingerprints implemented in Open Babel combined with the Tanimoto coefficient (Tc) as a similarity measure. For all tested methods, we computed enrichment factors (EFs) for 0.25%, 1%, 5% and 10% of the top ranked predictions. For every threshold used, DeCAF produced the highest enrichment factor (Table 1). The biggest improvement over fingerprints can be seen between 0.25 and top 1% of the ranking list. Although the difference is not large, it clearly showcases that 2D pharmacophore graphs with no predefined features can provide equal or even better performance.

**Figure 4:**
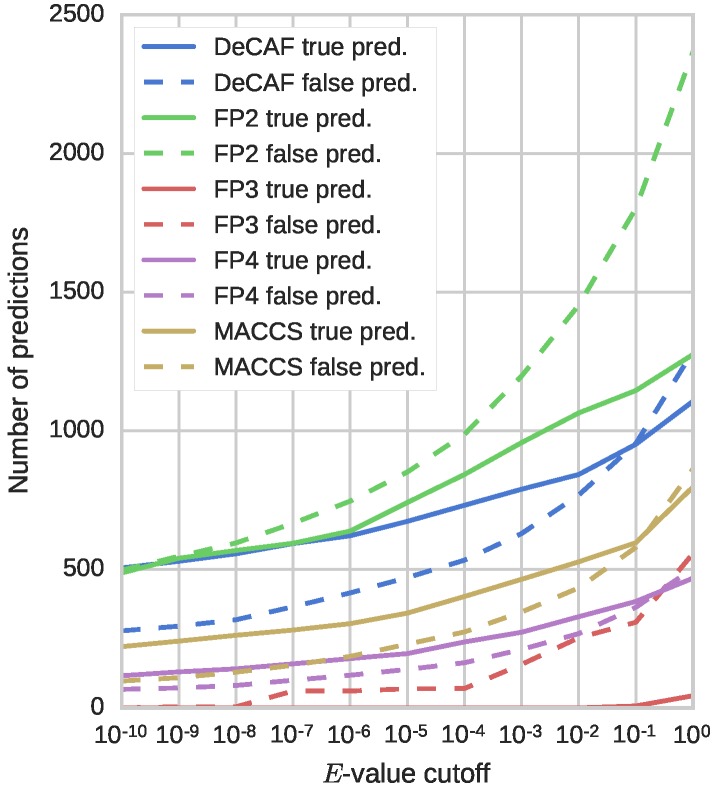
Reliability of the predictors. The plot shows the number of true (solid line) and false (dashed line) predictions with *E*-values below the specified cutoff.

The results from Table 1 show that overall performance of DeCAF, measured with ROC AUC values, is similar or better to the tested fingerprints (besides FP3, which performed substantially worse than other methods; the curves are presented in Supplementary Figure 1). The difference, however, is in the early enrichment, where DeCAF clearly shows its advantage. Further analysis of these results, presented in Figure 4, shows the number of true and false predictions as a function of the *E*-value threshold for each of the five tested methods. The best fingerprint (FP2) returns many more false predictions than DeCAF. Although FP4 and MACCS (Molecular Access System) keys provide fewer false predictions than FP2 or DeCAF, they also return fewer true predictions, especially with high confidence. Overall, DeCAF provides a high number of true positives with high confidence scores, along with a low number of false positives with low confidence score. Such feature combination is not available from any of the tested fingerprints.

#### 3.2.2. Comparison to USRCAT

USRCAT, a 3D method utilizing shape recognition, was used for comparison with DeCAF. We chose USRCAT as it compares molecules efficiently and the only time-consuming step is the multiple conformer generation process (see Section 1). Nevertheless, it was impossible to test USRCAT on our full benchmark because data preprocessing would be too expensive, especially when a statistical background is computed (see Supplementary Methods). Note, however, that if one already has precomputed ligand conformations, the USRCAT algorithm is highly efficient, comparable in speed to fingerprints.

USRCAT was originally validated on the DUD-E dataset and the results are freely available. However, as it was mentioned in the Section 2.3, using DUD-E would give DeCAF an unfair advantage (DUD-E decoys are generated as dissimilar structures; spatial structural features, however, are utilized by DeCAF). Therefore, DeCAF and USRCAT were compared on our dataset using targets with at most 1000 active ligands, yielding 26 small subsets.

As in the original publication, USRCAT was tested in two settings: (i) using up to 30 conformers (USRCAT_30); and (ii) using one, low energy conformer (USRCAT_1) of a molecule. The first test required much more computation, but provided better sampling, while the second was faster, but potentially less effective.

**Figure 5:**
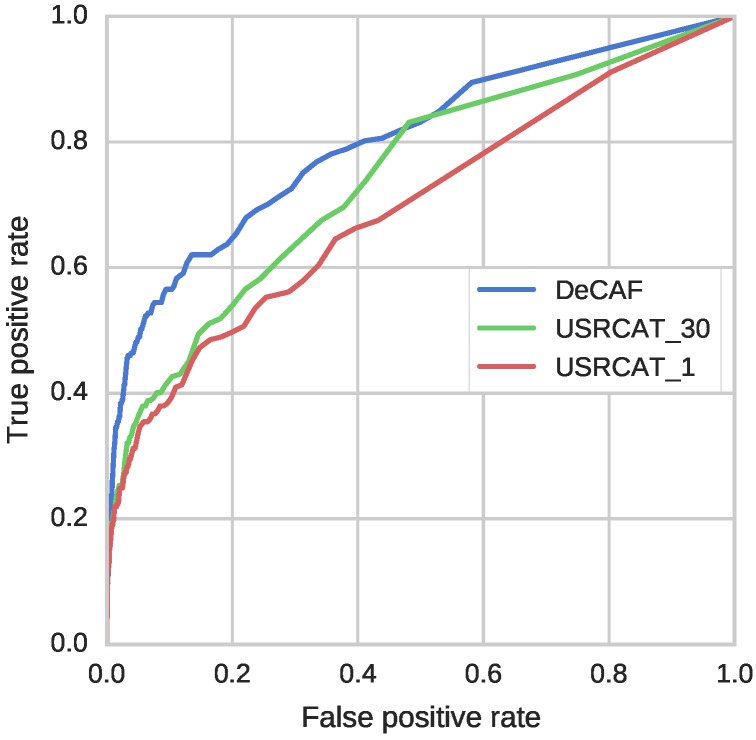
ROC curves for DeCAF and USRCAT. Areas under the curves are equal 0.80, 0.74, and 0.70 for DeCAF, USRCAT_1 and USRCAT_30, respectively.

The qualities of predictions were assessed using ROC curves and enrichment factors (Figure 5 and Table 2). In agreement with the results presented by Schreyer and Blundell, USRCAT performed very similarly when using a single conformer and 30 conformers. Note that, for the 0.25% threshold, the enrichment factor for USRCAT_1 was even higher than for USRCAT_30 and DeCAF. For all other fractions of the database, the highest EF and the highest ROC AUC values were obtained by DeCAF. Overall, DeCAF performance was comparable or better than USRCAT. It is also important to note the computation time needed for conformer generation, which is saved when using DeCAF. This allows assessment of much larger datasets, with many more potentially important targets.

## 4. Discussion

In this section, we focus on the results from the second benchmark, i.e., screening with an SEA classifier. This benchmark provides non-relative scores for activity prediction, with the same interpretation across different datasets and different representations of a molecule. This allows to directly compare activity predictions obtained with different methods.

In most cases, DeCAF’s advantages over the four fingerprints tested on SEA benchmark can be explained by the limited capabilities of these fingerprints to represent complex substructures and spatial relationships between different parts of a molecule (see Figure 4 in Supplementary Analysis). On the other hand, 3D methods such as USRCAT rely on the shape of a compound and therefore can suffer from using incorrect conformation(s). DeCAF tries to overcome those two contradicting approaches by converting a molecule into a 2D graph preserving the spatial relationships between pharmacophore groups. Although DeCAF performs generally better than the tested fingerprints, it sometimes fails to correctly predict activity when default parameters are used. Contrary to fingerprints, however, DeCAF allows to adjust feature importance and parameters of the alignment procedure. In this section, we show two examples that illustrate how a researcher can fine-tune DeCAF for a specific task. For more general assessment of the results, see Supplementary Analysis.

**Figure 6:**
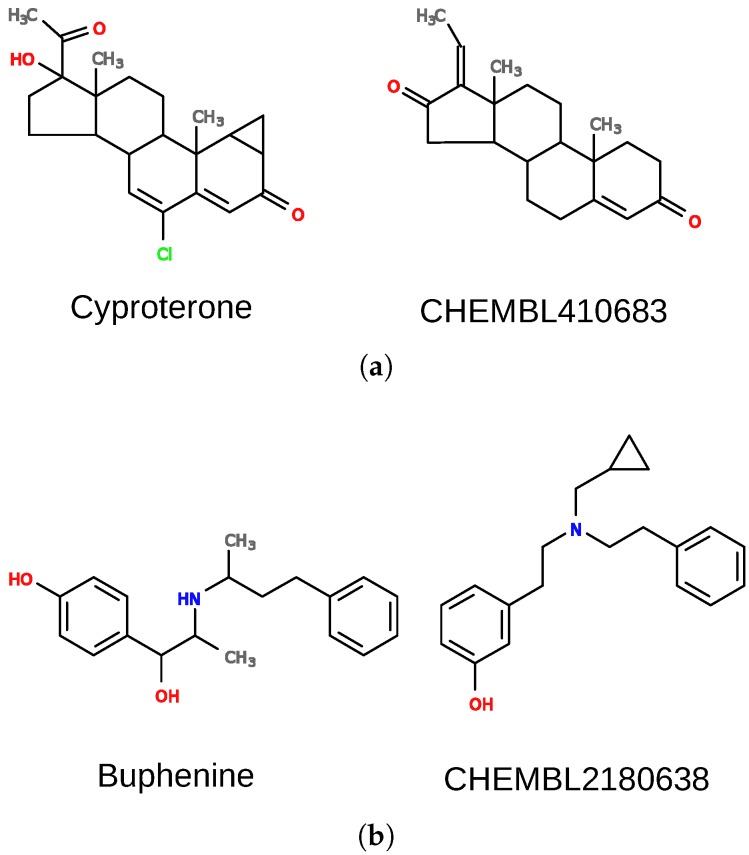
Examples of drugs (left) with incorrectly predicted activity and ligands of receptors (right) they were compared to. See Section 4 for more details. (**a**) cyproterone and guggulsterone (CHEMBL410683), a native ligand of pregnane X receptor; (**b**) buphenine and the native ligand of kappa-type opioid receptor.

The first case is a false positive result for pregnane X receptor (nuclear receptor subfamily 1 group I member 2; NR1I2). There is no evidence that cyproterone (Figure 6a) interacts with the receptor, yet it was predicted active, with high confidence (*E*-value of $5.25\times 10^{-21}$). Cyproterone shares a steroid scaffold with several pregnane X receptor ligands (e.g., CHEMBL410683), which is the biggest part of the molecule and dominates other features. There are, however, additional elements in the structure of cyproterone that probably prevent its interaction with the receptor, and they should also be important for the overall score. For such cases, the weights of some elements of the model can be changed to adjust their importance. When weights of nodes forming the ring system (with feature “R”) were decreased by a factor of 10 and other weights were increased by a factor of 10, the similarity of the two compounds dropped to 0.62.

Importantly, weights’ adjustment, as seen in the above example, may also be used in scaffold-hopping campaigns. In such cases, one would decrease the weight of specified scaffold atoms, and/or increase weights of ligands’ external pharmacophore groups responsible for target interactions. It is also possible to adjust the distance matching parameter, as shown below.

Another example is a case in which DeCAF fails to find true positives. Buphenine (Figure 6b) is clearly similar to certain ligands of the kappa-type opioid receptor (OPRK1), yet it obtains an extremely poor *E*-value of $4.96\times 10^{3}$ for interaction with this receptor. DeCAF returns low similarity scores for buphenine and other ligands of the kappa-type opioid receptor due to different distances between the two rings in their structures. In such a case, it would be beneficial to use a less stringent distance matching value while aligning these compounds. In order to change this default behavior, dist_tol parameter should be set to a value higher than 0. When dist_tol was set to 1.0, it was possible to match both rings in the structures, and obtain a similarity score of 0.73 for the molecules showed in Figure 6b. As a result, we were able to predict buphenine interaction with the receptor with an *E*-value = $2.6\times 10^{-1}$. Please note that modifying this parameter can be beneficial for specific receptors as they can obviously differ in ligand promiscuity and specificity. However, changing them globally for all receptors may result in higher false positives rates.

## 5. Conclusions

The results presented in this work demonstrate that DeCAF performance in a ligand-based screening benchmark was comparable or in most cases even better than the tested fingerprints and USRCAT.

When describing a molecule, DeCAF emphasizes ligand physicochemical properties and their relative arrangements. Such a model, by capturing the spatial relationships needed for receptor interaction, should allow for the identification of molecules with different structures but similar interaction patterns. Furthermore, DeCAF’s models and alignment procedure can be further adjusted and tailored for a particular research problem, dealing with specific ligand groups independently, as seen with the examples in the Section 4. To our knowledge, it is a unique and highly needed feature, which allows researchers to incorporate expert knowledge and gain insight into the computational model.

It is worth emphasizing that other applications besides this presented in this work are also possible. DeCAF can be used to create complex pharmacophore models by combining multiple chemical structures. Such models can also be adjusted to differentiate between crucial and insignificant features. Last but not least, DeCAF was implemented as a free, open-source Python package freely available at http://bitbucket.org/marta-sd/decaf. We also provide all data used in our study and Python scripts used to create and analyze them as a git repository at http://bitbucket.org/marta-sd/decaf-supplementary.

## Figures and Tables

**Table 1:** ROC AUC and enrichment factors for DeCAF and four fingerprints. EF values were calculated for 0.25%, 1%, 5% and 10% of top ranked predictions.

Method	ROC AUC	EF 0.25%	EF 1%	EF 5%	EF 10%
**DeCAF**	0.85	19.71	15.65	10.32	6.68
**FP2**	0.85	13.51	12.43	9.71	6.40
**FP3**	0.67	0.00	1.87	2.21	2.12
**FP4**	0.83	14.45	12.62	7.73	5.58
**MACCS**	0.82	17.08	13.92	9.53	6.09

**Table 2:** Enrichment factors and ROC AUC for DeCAF and USRCAT. EF values were calculated for 0.25%, 1%, 5% and 10% of top ranked predictions.

Method	ROC AUC	EF 0.25%	EF 1%	EF 5%	EF 10%
**DeCAF**	0.80	37.70	22.86	9.38	5.61
**USRCAT_30**	0.74	35.98	19.47	6.93	4.22
**USRCAT_1**	0.70	42.84	18.20	6.34	3.84

## Supplementary Materials

Supplementary materials are available online.

## Abbreviations

The following abbreviations are used in this manuscript:

DeCAF	Discrimination, Comparison, Alignment tool for 2D PHarmacophores
SEA	Similarity Ensemble Approach
USRCAT	Ultrafast Shape Recognition with CREDO Atom Types
CoMFA	Comparative Molecular Field Analysis
SMARTS	SMiles ARbitrary Target Specification
DUD(-E)	Directory of Useful Decoys (Enhanced)
MUV	Maximum Unbiased Validation
MACCS	Molecular Access System
Tc	Tanimoto coefficient
EF	Enrichment Factor
ROC	Receiver Operating Characteristic
AUC	Area Under Curve
