# Supplementary material for: DeCAF—Discrimination, Comparison, Alignment Tool for 2D PHarmacophores
Source: Molecules. 2017 Jul 6;22(7):1128. doi: 10.3390/molecules22071128 (PMC6152008; doi:10.3390/molecules22071128)
Supplement: Supplementary file 1 [file molecules-22-01128-s001.zip › Supplementary_Analysis.pdf]

Supplementary Analysis

1 Distribution of similarity scores

To better explain the differences between DeCAF and other methods tested SEA benchmark, we analyzed distributions of the similarity scores for a random probe of molecules (see Figure 1 and Table 1). Ligand-based virtual screening is based on the assumption that similar molecules have similar bioactivity. In order to be discriminative, a method should hardly ever yield high similarity scores for functionally unrelated, randomly chosen pairs of molecules.

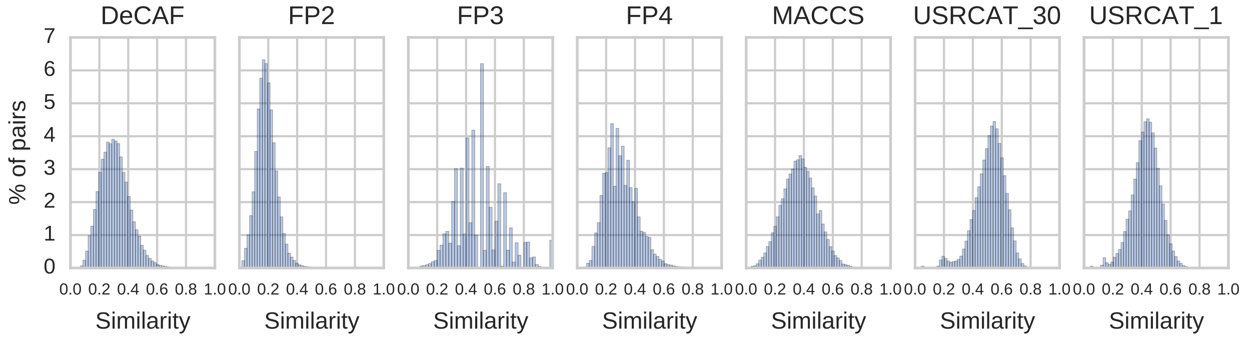

Figure 1: **Similarity score distributions for random data.** Each histogram was generated from the same random probe of molecules from the ChEMBL database.

Table 1: Properties of similarity score distributions

**Method**	**Mean**	**SD**	**% of scores exceeding**		
			**0.5**	**0.7**	**0.9**
**DeCAF**	0.307	0.101	3.945	0.113	0.004
**FP2**	0.188	0.070	0.130	0.011	0.000
**FP3**	0.492	0.169	49.602	12.429	1.769
**FP4**	0.304	0.111	6.070	0.241	0.005
**MACCS**	0.370	0.119	14.502	0.353	0.00
**USRCAT_30**	0.518	0.103	61.840	1.667	0.001
**USRCAT_1**	0.431	0.095	22.551	0.144	0.001

Our analysis shows that FP3 is an inadequate compound representation for predicting activity using similarity calculations (see Results in the main text). As shown in Figure 1, FP3 combined with the Tanimoto coefficient often returns high similarity scores for random pairs of molecules. The mean value of Tc for our random probe was 0.49, and almost 1.7% of pairs were considered identical (see example in Figure 2). There are only 55 features defined for FP3, which is significantly fewer than in other methods (MACCS, for example, uses 166 features). It seems that the 55 features in FP3 are insufficient to properly describe a molecule.

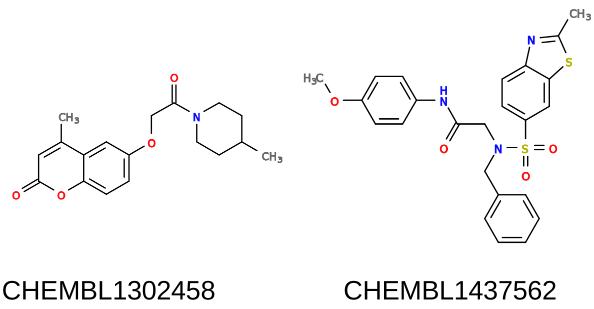

Figure 2: **Example of two molecules considered identical when represented with FP3.** FP3 is calculated using only 55 features, and it has very limited ability to describe a molecule.

2 Distribution of Z-scores

Our experiments show, that FP4, MACCS, and USRCAT perform adequately at discriminating interacting and non-interacting molecules, yet they return fewer results with high confidence than DeCAF. Figure 3 shows that the ranges of Z-scores (distance between the obtained score and the mean, expressed in standard deviation units) for interacting and non-interacting drug-target pairs are well separated; however, the difference between the ranges is very small.

All three representations return high similarity scores for random molecules more easily than DeCAF or FP2 (Figure 1 and Table 1). This translates into relatively low Z-scores for true predictions (Figure 3). Despite having only slightly lower enrichment factors than DeCAF, much lower Z-scores (or, equivalently, higher E-values) for true predictions makes FP4, MACCS, and USRCAT more difficult to use when solving real-life problems, in which true predictions are unknown.

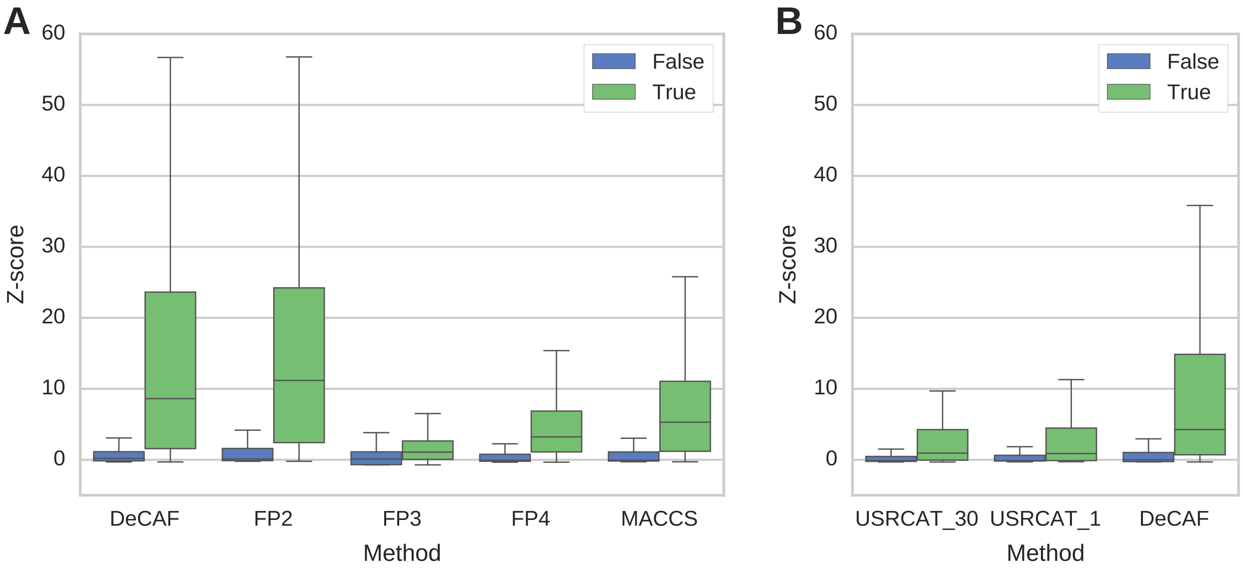

Figure 3: **Z-score distributions for the tested representations: DeCAF, 4 different fingerprints (A), and USRCAT (B).** Results for USRCAT were calculated on a smaller dataset (see "Comparison to USRCAT") using either a single or 30 conformers of a molecule (USRCAT_1 and USRCAT_30, respectively). The boxplots for false predictions (i.e., non-interacting drug-target pairs) are colored in blue and true predictions are in green.

When looking for candidate drugs that would interact with a given target, one needs to select a Z-score (or E-value) threshold to choose molecules that will be tested experimentally. This threshold is selected a priori, based on preliminary data or expert knowledge of the target and its known ligands. This selection must be performed very carefully, especially if the difference between Z-scores for interacting and non-interacting drug-target pairs is small. As a consequence, a small change in the threshold might yield many false positives (if

underestimated) or false negatives (if overestimated). In the cases of DeCAF and FP2, the Z-scores for true and false predictions differ considerably, and a small change in the threshold affects the results only slightly.

3 Incorrect predictions for FP2

In this section, we focus on FP2 because it performance on SEA benchmark better than any other tested fingerprint and USRCAT.

Although FP2 is able to predict many true drug-target interactions, it often returns false positives, especially for higher (but still reasonable) E-value thresholds (see Figure 2 in the main text). For FP2, which is a path-based fingerprint, the algorithm retrieves fragments of up to seven atoms and encodes them using a hash function. Those fragment are fairly small and FP2 might lose information about bigger substructures and their spatial arrangement.

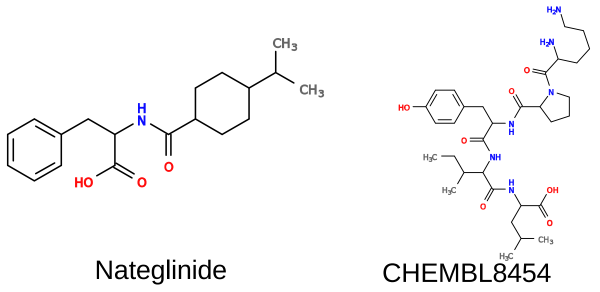

(A) **Nateglinide and one of the neurotensin receptor ligands.**

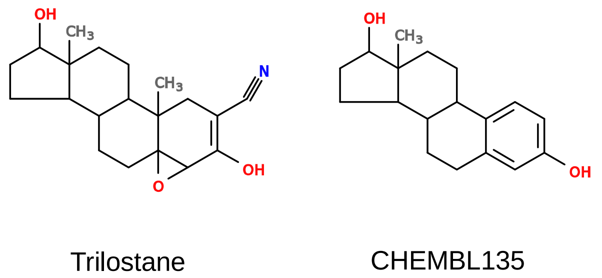

(B) **Trilostane and estradiol (CHEMBL135), two estrogen receptor ligands.**

Figure 4: **Examples of drugs (left) with incorrectly predicted activity and ligands of receptors (right) they were compared to.**

To illustrate this, let us refer to the example of neurotensin receptor type 1 (NTSR1, a member of the GPCR receptor family). One of the false positives found with FP2 is nateglinide (Figure 4A). Nateglinide shares some substructural features with many native ligands of NTSR1, but it is also substantially different, especially considering size and the peptide nature of NTSR1 native ligands. When represented as FP2, nateglinide and native CHEMBL8454 received a high Tc value of 0.72. At the same time, the similarity score calculated with DeCAF is 0.49. This is due to DeCAF's ability to represent the whole molecule regardless of its size and structure. FP2 (and other fingerprints used in this study) encodes only the presence or absence of features without any information about their spatial arrangement or number of times they appear. For presented NTSR1 ligand, there are 96 active bits (features) in FP2. On the other hand, nateglinide is characterised by 74 active bits, 68 of which occur in both compounds. Therefore, FP2 is unable to capture the complexity of NTSR1 ligands and describe them properly.

An overly simplified description of a molecule can also result in low similarity scores between evidently similar compounds. For example, trilostane can interact with the estrogen receptor (ESR1) and is highly similar to its bioactive ligands (Figure 4B). Nonetheless, Tc for trilostane and estradiol is 0.13 for FP2, which makes interaction between the ESR1 and trilostane impossible to predict. The steroid scaffold is poorly represented in FP2 as the ring system has far more than 7 atoms. However, DeCAF detects similarity between trilostane and other estrogen receptor ligands (similarity score for trilostane and estradiol is 0.85), predicting an interaction with high confidence (E-value of $1.0 \cdot 10^{-34}$).
