# Supplementary material for: DeCAF—Discrimination, Comparison, Alignment Tool for 2D PHarmacophores
Source: Molecules. 2017 Jul 6;22(7):1128. doi: 10.3390/molecules22071128 (PMC6152008; doi:10.3390/molecules22071128)
Supplement: Supplementary file 1 [file molecules-22-01128-s001.zip › Supplementary_Methods.pdf]

Supplementary Methods

1 DeCAF

1.1 Cycles in molecule representation

When converting a molecule to a pharmacophore model, DeCAF may produce cycles that do not correspond to rings in the molecule (Figure 1). This can occur when there is an atom in the molecule that is not included in the model. If such an atom connects three or more parts of a molecule, DeCAF adds edges between these parts to express distances between features, resulting in a cycle.

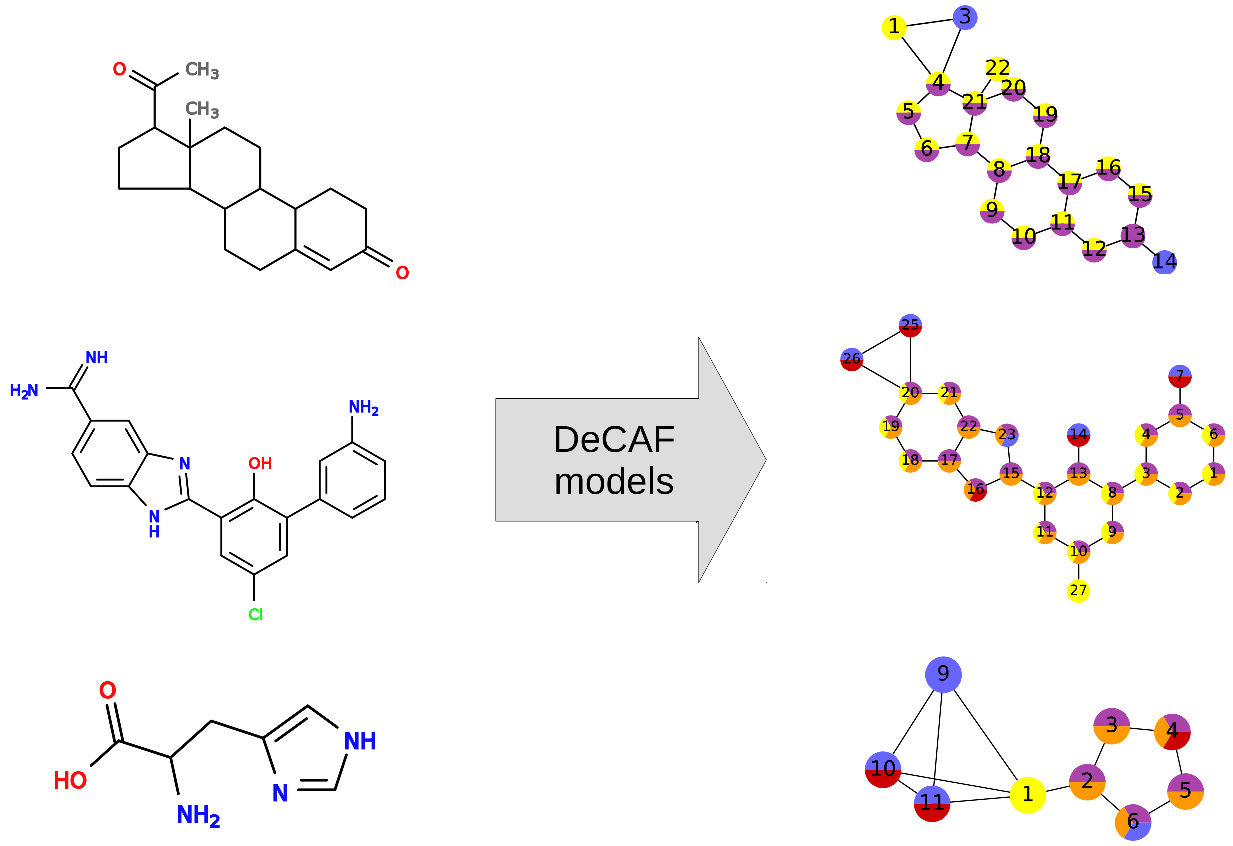

Figure 1: **Examples of cycles that do not correspond to rings in the molecule.** Note that the last graph cannot be depicted on a plane and thus the edge lengths in the figure do not match the edge weights in the model (i.e. edges between nodes 10 and 11 and between 10 and 9 have both weight 3).

For example, in the -CC(=O)OH fragment, the carboxyl carbon does not have any pharmacophoric features and is thus excluded from the model. As a result, DeCAF forms a cycle (triangle) composed of the oxygen atoms and the remaining carbon atom to represent this moiety.

DeCAF uses "R" feature to distinguish this type of cycles from molecular rings and they are not compressed during the first phase of the alignment procedure (see next section).

1.2 Models' comparison and merging

In order to compare and combine models, DeCAF finds alignment of the two graphs, i.e. their isomorphic subgraphs. To find common part of the two models, DeCAF generates their modular product and then searches for the highest-scoring maximal clique in this product.

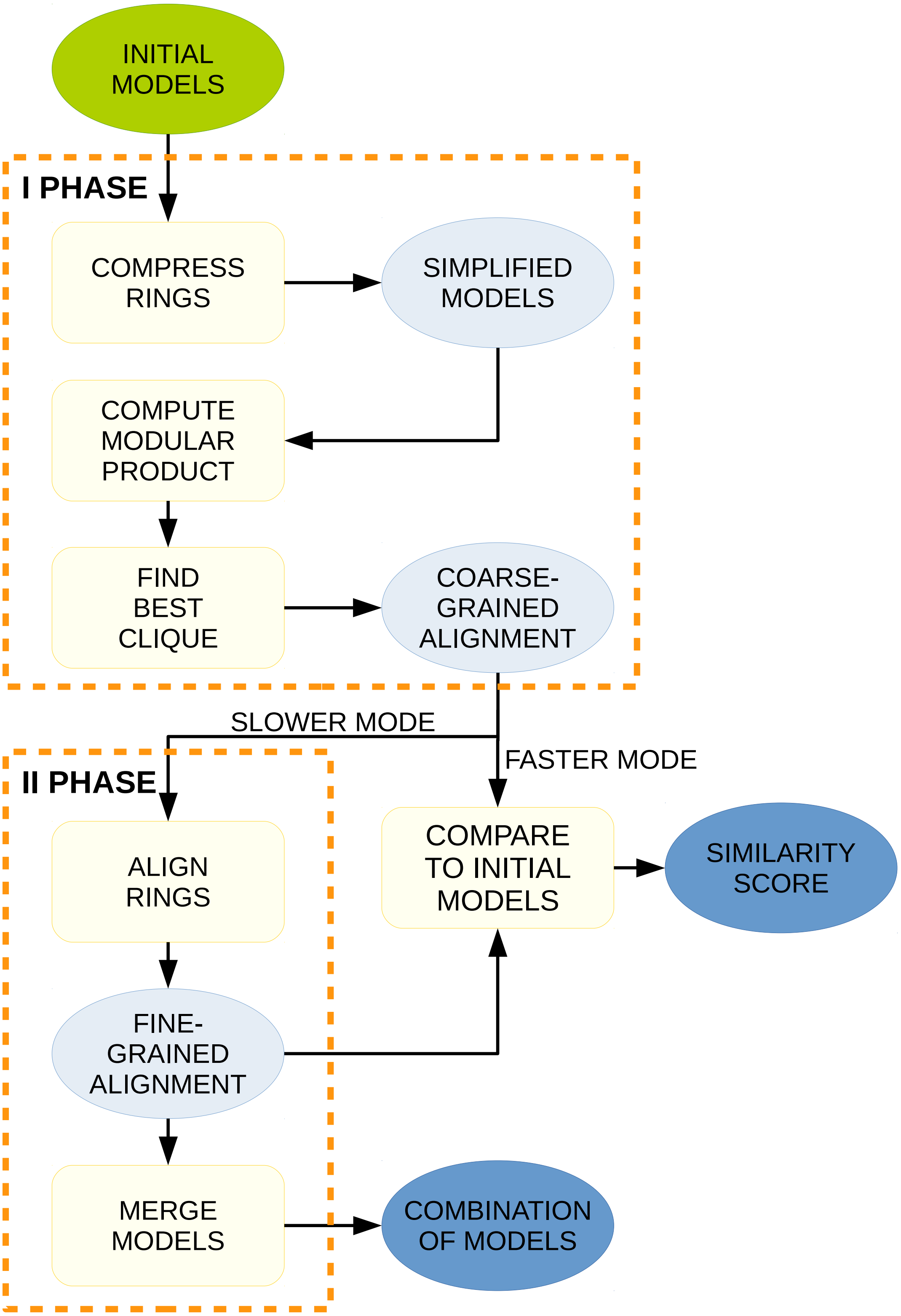

Figure 2: **DeCAF's alignment procedure.** Models are reduced for fast, coarse grained alignment. After the initial stage, ring systems are replaced by their uncompressed forms and a fully featured alignment procedure is applied.

Modular product – also called association graph – represents all possible common subgraphs (alignments) of two graphs, G and H. Let u and u' be the nodes from G and v and v' be the nodes from H. If nodes u and v can be matched, they constitute the node (u, v) in the modular product. An edge connects nodes (u, v) and (u', v') if either: u is connected to u' and v is connected to v', or both pairs (u, u') and (v, v') are disconnected. As a result, a clique in the modular product can be translated into an alignment between subgraphs of G and H.

Finding all maximal cliques (or, equivalently, the biggest clique) in the graph is NP-hard, therefore subgraphs isomorphism problem is NP-hard as well. In order to shorten computation, DeCAF constructs modular product with reduced number of nodes and edges and utilize standard, exponential-time algorithm to find cliques.

The algorithm is divided into two phases (Figure 2). In the first phase a coarse-grained alignment is produced. In the second phase, this alignment is transformed into a fine-grained alignment.

To reduce the models, rings and ring systems are compressed to single nodes using the pharmacophore "R" feature. By compressing rings, DeCAF decreases the number of nodes in the graph, which makes a great difference for exponential-time algorithms. Additionally, rings are often highly symmetrical, and it is possible to generate multiple equivalent alignments of two rings. The best alignment of the molecules is, therefore, defined by the arrangement of other features.

After ring compression, the modular product is generated. Two nodes $u \in G$ and $v \in H$ are considered compatible and form node (u, v) in modular product if they share any pharmacophoric features. The algorithm allow mapping part of a ring on a aliphatic fragment of a molecule, but disallows mapping parts of two rings to each other. This ensures that the rings are mapped to each other only in their compressed form, which shortens the computations.

In the next step, edges are added to the modular product. Accepted difference between distances is controlled by `dist_tol` parameter and by default it is set to 0 (i.e. by default edges $(u, u') \in G$ and $(v, v') \in H$ must have equal lengths). This additional requirement decreases the number of edges in the modular product and therefore reduces the number of cliques. It also prevents DeCAF from producing alignments that do not preserve distances between features.

After creating a modular product of two models, DeCAF finds all maximal cliques within it using the Bron-Kerbosch algorithm with pivoting. We select a clique with the highest score; however, it might not be the biggest clique because the nodes in a model may have different weights.

The resulting coarse-grained alignment is the optimal alignment of the reduced models and an approximation of the actual comparison of the two original models. Thanks to the ring compression, the coarse-grained alignment is generated very quickly. However, more importantly, it can be readily converted into an alignment with one-to-one correspondence between nodes from the original models, which is the purpose of the second phase of the alignment procedure. It can be also used to calculate approximation of a similarity score.

In the second phase of the algorithm, the alignment of non-ring atoms is copied from the coarse-grained alignment to the original models. Then all nodes that form a ring are aligned with respect to the already aligned parts of the pharmacophores (see Figure 3). The final alignment is locally optimal, i.e. it is the best fine-grained alignment that can be obtained with a given coarse-grained alignment as a starting point.

Fine-grained alignment can then be used to calculate the similarity between the two models (see next section) or to combine them into a single pharmacophore.

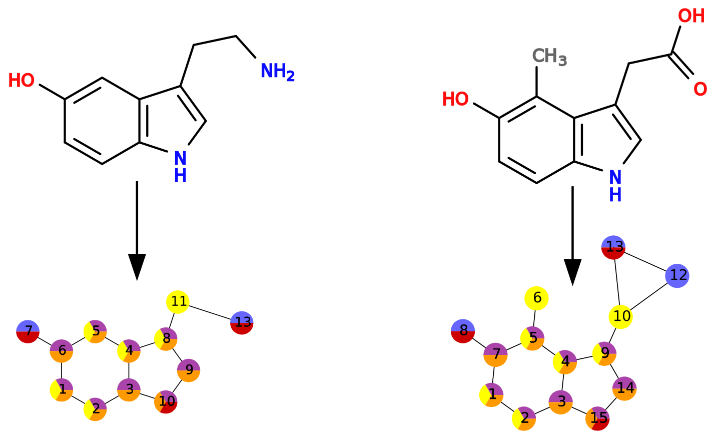

(a) **Create models.**

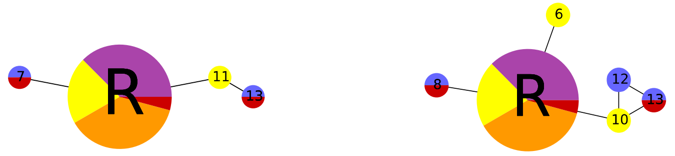

(b) **Phase I: Align models in their compressed forms.** Nodes correspondence: 7:8, 11:10, 13:13, -:6, -:12, R:R

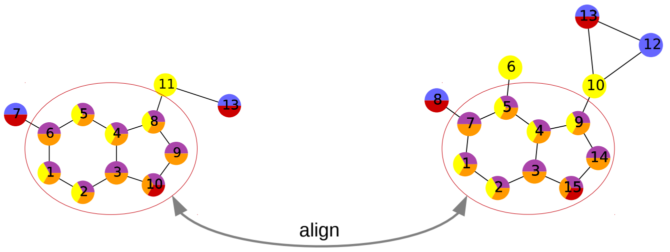

(c) **Phase II: Replace compressed nodes with original rings and align them.** Nodes correspondence: 7:8, 11:10, 13:13, -:6, -:12, 1:1, 2:2, 3:3, 4:4, 5:5, 6:7, 8:9, 9:14, 10:15

Figure 3: Alignment of two molecules.

2 Similarity Ensemble Approach

In the Similarity Ensemble Approach (SEA), each target is represented by a set of its ligands. To predict the activity of a drug against a given target, its overall similarity to the set of ligands is evaluated.

For each drug-target pair, we calculated the raw score, which is a sum of the similarity scores between the candidate molecule and all ligands of a given target that exceeds a specified threshold. For each threshold value (we used thresholds from 0.1 to 0.9 in 0.1 intervals) and each representation of a molecule, we found a relationship between the size of a random dataset and its average raw score (see next section).

Using these background distributions, we were able to calculate Z-scores (standard score; distance between the obtained score and the mean, expressed in standard deviation units) and E-values (expected number of results as extreme as the observed one for randomly distributed data) for each drug-target pair. Then, for a given drug-target pair, we selected the prediction with the lowest E-value (among nine of the used thresholds) as the final result.

2.1 Background distribution

We found relationship between raw score mean (μ) and standard deviation (σ) and dataset size (n) for every threshold:

$$\mu(n) = m \cdot n$$

$$\sigma(n) = s \cdot n^p$$

Values of m, s and p were estimated with 100 randomly selected sets of ligands (containing from 50 to 10000 ligands) corresponding to targets and 100 randomly selected molecules corresponding to drugs (see Table 1). The molecules were selected randomly from ChEMBL database (v. 20). The comparisons and parameters estimation were conducted with Python (see `http://bitbucket.org/marta-sd/decaf-supplementary`).

For USRCAT, which requires 3D structures, we used targets with up to 1000 ligands, and therefore estimated m, s and p using random samples with up to 1000 ligands as well. Also, because of the smaller random probes' sizes, it was impossible to model the relationship between dataset size and standard deviation of raw scores for similarity cutoff 0.9 – all but one raw score were equal to 0. We therefore excluded this cutoff from the procedure of comparing DeCAF and USRCAT.

We used those values to predict expected mean and standard deviation for random comparison for each target in our data. Size- and threshold-dependent parameters were use to calculate Z-scores:

$$z = \frac{x - \mu(n)}{\sigma(n)}$$

Then, under the assumption that Z is from Gumbel distribution (also called "extreme value distribution"), we calculated p-values and E-values for every prediction:

$$p(z) = P(Z > z) = 1 - exp(-e^{\frac{-\pi z}{\sqrt[2]{6}} - \gamma})$$

$$E(z) = p(z) \cdot N$$

where γ is Euler-Mascheroni constant and N is a number of comparisons made ($N = 73 \cdot 656 = 47888$).

Table 1: Parameters' estimates

		**Threshold**								
**Parameter**	**Method**	**0.1**	**0.2**	**0.3**	**0.4**	**0.5**	**0.6**	**0.7**	**0.8**	**0.9**
	DeCAF	0.3035065	0.2797780	0.1895935	0.0794863	0.0210566	0.0041835	0.0007247	0.0001260	1.521e-05
	FP2	0.1810923	0.1013965	0.0207360	0.0035608	0.0007888	0.0002159	6.499e-05	2.263e-05	6.370e-06
	FP3	0.4924598	0.4902398	0.4696895	0.4025631	0.3115910	0.1816387	0.0993094	0.0560340	0.0177328
**m**	FP4	0.3030035	0.2785899	0.1888104	0.0894460	0.0324056	0.0076763	0.0015139	0.0002751	3.767e-05
	MACCS	0.3701049	0.3592636	0.3066053	0.1968739	0.0826292	0.0195726	0.0026330	0.0002164	1.623e-05
	USRCAT_1	0.4299535	0.4272054	0.4094915	0.3140922	0.1220590	0.0187504	0.0010025	1.266e-05	
	USRCAT_30	0.5159234	0.5143510	0.5079490	0.4768428	0.3524359	0.1291256	0.0114793	0.0001123	
	DeCAF	0.0502145	0.0742986	0.0829726	0.0492985	0.0191615	0.0066157	0.0020309	0.0012475	0.0008889
	FP2	0.0403341	0.0474950	0.0169255	0.0048703	0.0013854	0.0009414	0.0011044	0.0011975	0.0004909
	FP3	0.0784411	0.0822976	0.1042186	0.1315044	0.1309848	0.1015750	0.0710657	0.0514703	0.0243483
**s**	FP4	0.0534027	0.0729263	0.0891716	0.0641477	0.0319038	0.0115124	0.0038517	0.0012729	0.0015229
	MACCS	0.0569994	0.0677961	0.0908296	0.0940940	0.0617143	0.0221173	0.0044358	0.0019466	0.0007762
	USRCAT_1	0.0592863	0.0739646	0.0891587	0.1190982	0.1003300	0.0338040	0.0093708	7.189e-06	
	USRCAT_30	0.0657212	0.0736884	0.0909817	0.1135708	0.1517178	0.0919997	0.0248735	0.0053312	
	DeCAF	0.9996262	1.0000292	0.9996615	0.9978054	0.9937496	0.9826808	0.9593771	0.8380675	0.6691711
	FP2	0.9999422	1.0005044	1.0035328	0.9911516	0.9848265	0.8913990	0.7600372	0.6677050	0.6786393
	FP3	0.9990369	0.9990742	0.9986642	0.9974278	0.9975485	0.9998821	1.0025588	1.0042265	1.0031570
**p**	FP4	0.9967858	0.9964624	0.9966353	0.9972864	0.9975567	0.9893839	0.9609965	0.9078803	0.7003041
	MACCS	0.9998887	0.9997813	0.9989737	0.9993850	0.9996967	0.9992509	0.9854968	0.8359487	0.7012564
	USRCAT_1	0.9879909	0.9823904	0.9952173	0.9907030	0.9747262	0.9342572	0.7692190	1.4051754	
	USRCAT_30	1.0041271	1.0004813	1.0038453	1.0075073	1.0032742	1.0024363	0.9059835	0.6042358	
