# Supplementary figures and images for: DeCAF—Discrimination, Comparison, Alignment Tool for 2D PHarmacophores
Source: Molecules. 2017 Jul 6;22(7):1128. doi: 10.3390/molecules22071128 (PMC6152008; doi:10.3390/molecules22071128)

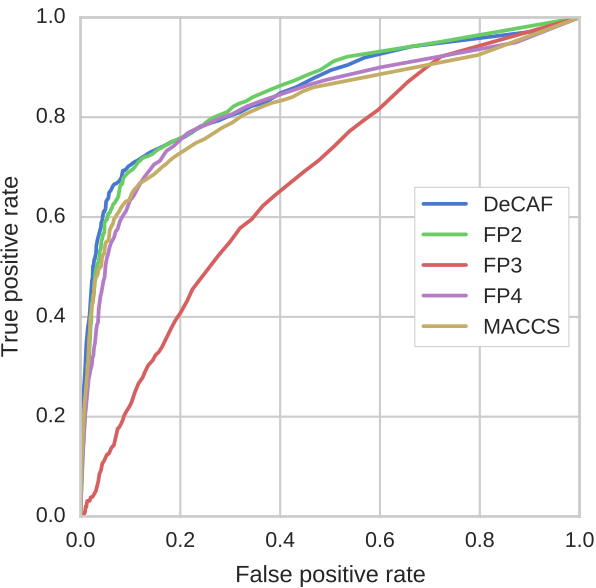

Supplement: Supplementary file 1 [file molecules-22-01128-s001.zip › Supplementary_Figure_1.pdf]
